# Guest editors’ introduction: special issue “ICT in language learning”

**DOI:** 10.1186/s41039-015-0025-x

**Published:** 2015-11-14

**Authors:** Yu-Ju Lan, Ting-Chia Hsu

**Affiliations:** 1 The Department of Applied Chinese Language and Culture, National Taiwan Normal University 162, Section 1, Heping E. Rd., Taipei City, 106 Taiwan; 2 Department of Technology Application and Human Resource Development, National Taiwan Normal University 162, Section 1, Heping E. Rd., Taipei City, 106 Taiwan

Owing to the rapid development of information and computer technology, numerous studies have investigated how to harness state-of-the-art technologies for effective language teaching and learning in the past decades. The unique features of modern information and communication technologies (ICT), such as 3D virtual environments, mobile computing, embodiment, and visual learning, have been expanding the potential and possibility of promoting the idea of learning languages anywhere and anytime in immersive and interactive contexts. Language learning is no longer limited in traditional settings or approaches. With the usage of modern advanced technologies, language learning can be different experiences as we have so far. However, it is possible to encounter challenges and problems while introducing powerful learning technologies into practical application.

This special issue aims at providing a platform for researchers to present their efforts on studies that may offer insights into the practical and technical challenges that might be faced while applying advanced ICT technologies to language teaching and learning (LTL), and also aims at addressing important research trends and societal needs. It is expected that through the publication of this special issue, we can help develop a further understanding of ICT in LTL. After a rigorous review process, three high-quality research papers have been accepted for publication in this special issue. These papers clearly provide perspectives from different angles to the above concerns. We hope that these studies will inspire future research in this direction.

In the first paper entitled “The Effects of an online student question-generation strategy on Elementary school student English learning,” Yu, Chang, and Wu investigated the effects of employing the strategy of students generating questions via the support of an online system on learners’ English performance and learning motivation. A total of 106 sixth graders participated in this study. After analyzing the collected data, the authors found that the participants in the question-generation group significantly outperformed their peers without using the strategy. In addition, participants’ learning motivation was also enhanced by the support of the online system. In the second paper with the title of “GPS sensor-based mobile learning for English: An exploratory study on self-efficacy, self-regulation and student achievement,” Sun, Chang, and Chen built a GPS sensor-based mobile learning system for college students to learn about the plants in the campus in English in the real context. A total of 41 college students participated in this study. They were grouped into two groups according to their self-efficacy and regulation, high versus low in the two learner variables. After the experiment, although the participants, regardless originally in the high or the low group, made no significant improvement in self-efficacy or regulation, they made improvement in the learning achievement. The participants also thought that the context-aware mobile learning system was easy to use and was useful. Last but not least, in the third paper entitled “Using a tablet-based composition marking recording system to conduct think-aloud for composition rating research,” Leung, Mak, and Leung developed a tablet-based recording system to record the rating process of teachers teaching Chinese writing in a camera-free environment to successfully conduct the think-aloud protocol analysis in the Chinese composition rating process. By using the system, the rating process employed by the teachers of Chinese writing can be recorded objectively and further analyzed to better understand what dimensions and focuses a teacher of Chinese writing concerns in a Chinese composition.

The abovementioned papers will likely provide readers with a deep and extensive understanding of ICT in language learning. The covered target languages include English and Chinese. Moreover, the participants were drawn from a range of ages and roles, both elementary school students and college students and both students and teachers. The investigated variables relating to language learning are various, including achievement, motivation, self-regulation, and self-efficacy. Although the papers included in this special issue have covered a broad range of issues in ICT in language learning, there are other issues that may further attract researchers’ attention in the future. For example, serious game for digital natives’ language learning and learning analytics in language learning are two emerging issues that cannot be ignored in the big data era. Researchers’ efforts on exploring the potential of ICT in language learning will be worthwhile because ICT presents a potential solution to the problems encountered in today’s language education.

